# Analysis of microbiome diversity in coprolites from Caral, Peru

**DOI:** 10.6026/973206300181159

**Published:** 2022-12-31

**Authors:** Jaramillo-Valverde Luis, Váaacute;squez-Domínguez Andréeacute;s, Kelly S Levano, Novoa-Bellota Pedro, Machacuay-Romero Marco, Garcia-de-la-Guarda Ruth, Castrejon-Cabanillas Rony, Palma-Lozano Diana, Ashok K Sharma, Davison Samuel, Flores-Villanueva Pedro o, Cano Raul J, Gomez A, Shady-Solis Ruth, Guio Heinner

**Affiliations:** 1 ALBIOTEC, Lima, Perú; 2 INBIOMEDIC Research and Technological Center, Lima, Perú; 3 Universidad de Huánuco, Huánuco, Perú; 4 Facultad de Ciencias Biolóoacute;gicas, Universidad Nacional Mayor de San Marcos, Lima, Perú; 5 Zona Arqueológica Caral, Unidad Ejecutora 003, Ministerio de Cultura., Lima, Perú; 6 Escuela Profesional de ArqueologÍa, Facultad de Ciencias Sociales, Universidad Nacional Mayor de San Marcos, Lima, Perú; 7 Laboratorio de MicrobiologÍa Molecular y BiotecnologÍa, Facultad de Ciencias Biológicas, Universidad Nacional Mayor de San Marcos, Lima, Perú; 8 Department of Animal Science, University of Minnesota, St. Paul, MN; 9 The BioCollective, Denver, CO 80014, USA; 10 Centro de Investigación en Biodiversidad para la Salud, Universidad Privada Norbert Wiener, Lima, Perú; +These authors contributed equally to the article

**Keywords:** Microbiome, coprolites, caral and diversity

## Abstract

We analyzed human coprolites from the Sacred City of Caral, the oldest civilization in America (3000- and 1800-years BC). Our objective was to know the microbial diversity of the Caral Civilization through the use of a mobile ancient laboratory. DNA extraction conducted in a mobile laboratory placed near the collection site to reduce exposure of samples to contaminants and favor a rapid molecular processing. Using 16S rRNA and ITS 1 amplicon sequencing, we have elaborated the first list of the microbiomes of Caral, based on the bacterial and fungal community fingerprints detected in the coprolites recovered in six sectors of that ancient urban center. Among the most abundant sequences were those associated with Firmicutes for bacteria, Ascomycota and Basidiomycota for fungi. Bacillus was the most abundant bacterial genera in all samples analyzed, compromising up to 24.81% of the total bacterial abundance; while Aspergillus (11.43%) was the most abundant genera among fungal communities.

## Background:

Coprolites are desiccated fecal material preserved over time that can be found in archaeological settings where conditions are suitable for its preservation [1].These coprolites offer the potential to obtain unique knowledge about certain aspects of biology,ecology and behavior of prehistoric human and animal populations [2]. Such knowledge, often unattainable by studying other types of remains; provides details on dietary habits, agricultural practices, seasonal migration, health, and the state of an individual's intestinal microbiota, which would allow a deeper understanding of ancient civilizations. Throughout history, coprolites dating from the Paleozoic Era have been found [3], as have dinosaur coprolites from the Cretaceous period ca.145-66 Ma [4]. Analysis of microbiomes from ancient coprolites date from the beginning of the 21st century, where research groups amplified a portion of the 16S rRNA gene, and were able to find sequences that were generally consistent with the families and genera expected for intestinal bacteria, such as the case of Alpha-, Beta-, Gamma proteobacteria,Clostridium, Eubacterium, and Bacteroides species [5-9].The Sacred City of Caral, is located at the beginning of the middle sector of the Supe valley, Barranca province, 182 km north of Lima, in the north-central area of Peru. It is the most outstanding urban settlement of the Caral civilization for its extension and architectural complexity of all those identified in the New Continent between 3000- and 1800-years BC. Caral developed almost simultaneously with the civilizations of Mesopotamia, Egypt, India and China [10] The Sacred City extends 66 hectares, in which there is a nuclear and a peripheral sector. The first shows 32 monumental architectural structures,two classes of distinctive residential complexes, plus servants' households and storage units, two sunken circular plazas, and massive public congregation spaces [10]. The ancient inhabitants of the Caral civilization lived in nucleated settlements of various sizes, distributed throughout the valley, from the coast to the end of the middle valley; sustained by a self-sufficient economy. In the high Andean areas, the settlers were generally hunter-farmers; in the valleys of the Sierra, they were farmers-hunters and on the coast, they were fishermen, mollusk collectors and farmers [11]. Agro-fishing complementarity was fundamental for the subsistence of the Caral Civilization since its authorities extended the interregional and long-distance exchange [10, 12]. Because ancient DNA is most often present in trace amounts in archeological samples, contamination by modern or previously amplified DNA is a matter of concern. With this limitation in mind, we attempted to obtain the proof-of-concept for an ancient DNA mobile laboratory by focusing on DNA from coprolites of ancient inhabitants of the Caral Civilization; we take as a reference the study carried in France [13]. We report here on the use of a mobile laboratory consisting of commercially available devices for DNA extraction.Therefore, it is of interest to develop new methodologies that can be applied to identify possible coprolite microbiome fingerprints in archaeological centers in Peru.

## Materials and Methods:

## Samples:

To analyze the fecal microbiomes of the ancient inhabitants of the Caral, coprolite samples were used. 34 coprolites were collected from eight selected sectors of the Sacred City of Caral, and results were obtained for six sectors (Figure 1A-see PDF). In each sector, separated by an average of 200-500 meters, and differentiated by functionality by archaeologists, three to five samples were collected. To avoid the transit of people other than researchers, the areas to be sampled were delimited by a yellow tape.

Coprolites were collected following the procedure reported by Wood and collaborators [14], who described a procedure for sub-sampling coprolites to reduce the risk of contamination. Likewise, the protocols developed by Cone [15] were followed for the handling of coprolite samples in the laboratory.

For the sample collection process, the staff used sterile, personal protective equipment for each sector, to avoid added contamination by collectors. In the same way, the use of materials for the excavation (bumps, brushes, tweezers, among others) was used once in each sector. Three persons were required for the identification and extraction of coprolites: (1) The excavator was in charge of locating the coprolite samples, then with the help of a mason trowel, they pointed without touching the sample, (2) The collector approached the sample carefully avoiding lifting the dust as much as possible, collected the sample with the disposable forceps and placed it in a sterile bag, closing it immediately. (3) Information on sample collection was labeled on the sterile bag which was transported in a cooler immediately to the mobile laboratory to start the extraction DNA process.

## Sample processing and DNA extraction:

A mobile laboratory was designed and implemented, on-site, to perform DNA extraction of each coprolite sample. This mobile lab station contained all the necessary equipment, including a laminar flow cabinet, biosafety cabinet, water bath, microcentrifuge,vortex, refrigerator and freezer as well as air conditioning (Figure 1B -see PDF), thus, allowing us to process samples within 5 minutes after collection, and minimizing the risk of contamination, a common hurdle in ancient DNA analyses [13]. During extraction,each coprolite sample was placed in a Pyrex cabinet and irradiated with ultraviolet light for 20 minutes (both sides); subsequently, with a brush and scalpel (sterile), the central part of the coprolite was cutand separated. The subsample was again subjected to UV irradiation for 20 minutes and 250 mg of the coprolite was weighed into a microcentrifuge tube. The PowerSoil DNA Isolation Kit (QIAGEN) was used to purify DNA in the samples, including modifications such as the inclusion of incubation with 1x PBS at 4°C for 72 hours before extraction and incubation at 70°C for 10 minutes with the lysis solution C1 of the kit [16]. This modified protocol is reported to optimize and obtain better-quality ancient DNA, for Next Generation Sequencing (NGS) procedures [17]. To confirm the presence of DNA in the extracted samples, electrophoresis was performed on a 1% agarose gel and a 1 Kb marker (Invitrogen) was used as a reference. Ethidium bromide staining was used and visualized using a gel photodocumenter (Chemidoc, BIORAD). Additionally, samples that were noted to have DNA underwent gel purification with the DNA cleanup kit (QIAGEN), which further eliminated and removed impurities in the ancient DNA sample.

## Microbiome analyses from purified DNA:

To characterize the microbiome in the purified DNA samples, bacterial diversity was identified using the V4 variable region of the 16S rRNA bacterial gene (bacteriome), using primers 515F (GTGYCAGCMGCCGCGGTAA) and 806R (GGACTACNVGGGT WTCTAAT) [18]. Likewise, for the identification of fungal diversity (mycobiome), the analysis of the intergenic region (ITS 1) was carried out using the primers ITS1 (CTTGGTCATTTAGAGGAAGTAA) and ITS4 (GCTGCG TTCTTCATCGATGC) [18]. The construction and sequencing of the libraries were carried out at MR DNA Laboratory (www.mrdnalab.com, Shallowater, TX, USA) [18], on the Illumina MiSeq platform and using the bTEFAP method, which is a universal, high-throughput tool widely used for epidemiological and diversity pathogen studies. We aimed for a total of ~ 20K reads (2x300 PE) per sample for both 16S rRNA and ITS amplicons. Sequences less than 150 bp, with ambiguous bases and with homopolymer runs greater than 6 bp were eliminated, using a free access software www.mrdnafreesoftware.com [18]. Sequences were processed in the Qiime2 bioinformatics platform [19], to filter out low-quality sequences, and chimeras, assign taxonomic identity and generate tables of frequency and abundance of fungal and bacterial Operational taxonomic units (OTUs), defined by binning at 97% rRNA sequence similarity [19]. OTUs were taxonomically classified using BLASTn against a database derived from RDPII, which is specific for the analysis of ribosomal sequence markers (http://rdp.cme.msu.edu). Statistical analyses were performed using various packages in the R statistical software, including alpha and beta diversity analyses for all samples. analysis respectively. The relative abundance (%) at the phylum level is shown.

## Results:

## Bacterial and Fungal identification

Out of the 34 coprolite samples collected, 18 ancient DNA samples were selected based on purity to carry out the sequencing of bacterial (16S RNA) and fungal (ITS) diversity. Figure 2(see PDF) shows the taxonomic distribution of all samples, at the phyla level, for 16S rRNA and ITS data, with the phylum Firmicutes being the most prevalent across all samples (73.94%) followed by Proteobacteria (13.98%) (Figure 2a - see PDF). Regarding fungal diversity, the phylum Ascomycota constituted the most prevalent of all the fungal sequences found (59.61%), followed by Basidiomycota (38.50%) (Figure 2b - see PDF). Table 1(see PDF) shows the most abundant taxa in the dataset at the genus level, revealing that the most prevalent bacterial taxa found were Bacillus sp. (24.81%), Lentibacillus sp. (21.66%) and Tuberibacillus sp. (13.34%); in the case of fungi, the data show a higher prevalence of Aspergillus sp. (11.43%). Fungal diversity at the level of genera and OTUs was found to be greater than the diversity of bacteria in all the samples analyzed, according to the Shannon alpha diversity index (Figure 3a - see PDF). Furthermore, bacterial and fungal richness and diversity showed significant fluctuations in every sample analyzed according to the rarefied number of OTUs (20K reads) and the Shannon index of alpha diversity (Figure 3b - see PDF); thus, samples were highly heterogeneous as far as alpha diversity, which points out to presence and dominance of specific taxonomic groups in particular sites.

Taxonomic heterogeneity across samples can be seen in Figure 4(see PDF). For example, sample AB001 was largely dominated by an unidentified bacterial OTU of the genus Bacillus and by an unidentified fungal OTU. In contrast, sample AB-002, which belongs to the same sector as AB-001 (see map in Figure 1 - see PDF) shows a completely different, and more diverse taxonomic profile. Taxonomic variation was evident across all samples; however, as far as the bacteriome, most samples showed varying prevalence of diverse OTUs affiliated to the Bacillales and Actinomycetales orders (Figure 4a - see PDF). The mycobiome was also highly heterogeneous across all samples, but in contrast with the bacteriome, it exhibited a more diverse taxonomic distribution, highlighting presence and dominance of diverse OTUs from the several families such as Aspergillaceae, Ascobolaceae, and Cortinariaceae, among many others (Figure 4b - see PDF).

An analysis of beta diversity (Bray-Curtis distance matrix-) corroborates highly heterogeneous taxonomic patterns in bacterial and fungal diversity across samples, showing no evidence that the samples cluster by sectors (A, E, G, H, I, L) (Figure 5 - see PDF) or that the microbiome (both bacteriome and mycobiome) is similar depending on the site where the sample was collected.

## Discussion:

Regarding the taxonomic analysis; the phylum Firmicutes was found in great abundance in all samples; likely dominated by B acillus sp. The presence and frequency of the phylum Firmicutes, in the coprolite samples, in our case in low quantities, plays an important role in the Healthy Gut Microbiota Composition [20]. In addition, some researchers have determined that, if there is the presence of species such as Prevotella, is an indication of traditional and ancient microbiomes [21]. Since they were detected in large proportions, Ascomycetes and Basidiomycetes also appear to have been important dietary elements of these cultures. Although these conclusions are highly hypothetical and perhaps speculative, we believe this is a good starting point if we are to compare future studies such as the one carried out here. In the case of Aspergillus, this taxon can be found in the human gut [22] but are much more commonly reported in environment (soil, air, plant matter) than in gut samples, and are presumably of environmental origin [23-24], so it is difficult to draw a conclusion about this high prevalence. The observed differences, related to alpha diversity in fecal microbiota, probably suggest major variations in the diets of the inhabitants of the ancient urban center. This is further supported by the diversity and relative abundances of bacteria detected in each sector of the city, which hosted different activities, according to archaeological investigations. We report highly heterogeneous taxonomic patterns in bacterial and fungal diversity across samples, showing no evidence that the samples cluster by sector of the ancient city. We can point to sectors E and G (Mayor and Minor pyramid) as the less diverse in terms of bacteriome and mycobiome. These variations observed in the fecal microbiota, related to cultural and dietary differences, will be explored in future publications. The present study has some limitations that should be considered for future studies. DNA extraction controls for instance, from the surrounding soil to delimit only the microorganisms within the coprolite and that belong to the human intestine [25]. Also, some research reports the percentage of microorganisms that come specifically from human coprolites and animals such as Canis familiaris that lived for 14,000 years [26-28]. However, it has been reported that for diet studies the dog's microbiota can be a good proxy to determine the diet of the ancient population [29-30].

## Conclusion:

We successfully extracted and sequenced DNA from archaeological fecal samples in order to assess possible differences in the fecal communities of individuals from the Caral Civilization. Our data show that, contrary to common belief, the formation and preservation of coprolites and DNA contained in these coprolites under difficult environments for thousands of years is possible. This study is one of the first in its kind and we hope will point to the importance of coprolites as important cultural markers and thus any archaeological dig should include the search and preservation of any coprolites found at the sites. This study underlines theimportance of such samples for future paleomicrobiological studies. The results confirm that coprolites are not completely degraded in environments and thus can be formed under suitable taphonomy conditions. Finally, the samples were highly heterogeneous as far as alpha and beta diversity, which points to the presence and dominance of specific taxonomic groups in particular sectors.

## Financial support:

This work was funded by CONCYTEC-FONDECYT within the framework of the call E043-2018-FONDECYT/BM-IADT-AV and 19-2019-BM-INC.INV.

## References

[1] Lisa-Marie S (2020). Earth-Science Reviews.

[2] Bouchet F (2003). Memorias do Instituto Oswaldo Cruz.

[3] Dentzien-Dias PC (2013). PLoS One.

[4] Chin K (1998). Nature.

[5] Ubaldi M (1998). American Journal of Physical Anthropology.

[6] Cano RJ (2000). American Journal of Physical Anthropology.

[7] Poinar HN (2001). Proceedings of the National Academy of Sciences.

[8] Luciani S (2006). American Journal of Physical Anthropology.

[9] Rollo F (2007). Journal of archaeological science.

[10] https://www.zonacaral.gob.pe/.

[11] Shady R (2005). Investigaciones Sociales.

[12] https://revistaspucp.edu.pe.

[13] Utge J (2020). PLoS ONE.

[14] Wood JR, Wilmshurst JM (2016). Quaternary Science Reviews.

[15] Cone RW (1993). Genome Research.

[16] https://www.qiagen.com/us.

[17] Uquillas JA (2011). Biomedical Materials.

[18] Santiago-Rodriguez TM (2017). Genes.

[19] Rideout JR (2014). Peer J.

[20] Rinninella E (2019). Microorganisms.

[21] Gomez A (2019). Msphere.

[23] Zhang L (2021). Therapeutic Advances in Gastroenterology.

[24] Mortensen KL (2010). Antimicrob Agents Chemother.

[25] Pedersen MW (2015). Philos Trans R Soc B Biol Sci.

[26] Shillito LM (2020). Sci Adv.

[27] Rampelli S (2021). Science.

[28] Wibowo MC (2021). Nature.

[29] Witt KE (2021). Sci Rep.

[30] Jacobson DK (2020). Phil. Trans. R. Soc. B.

